# Validation of a Mass Spectrometry–Based Proteomics Molecular Pathology Assay

**DOI:** 10.1016/j.mcpro.2025.101487

**Published:** 2025-12-12

**Authors:** Jessica R. Chapman, Jeeyeon Baik, Oana Madalina Mereuta, Sansan Yi, Ayal Cooper Walland, Kristel Flor, Ashley Wooten, Jessica Wardrope, Maria Stella Ritorto, Ahmet Dogan

**Affiliations:** Department of Pathology and Laboratory Medicine, Memorial Sloan Kettering Cancer Center, New York, USA

**Keywords:** FFPE tissue, clinical, amyloidosis, validation

## Abstract

Mass spectrometry–based proteomics has been applied to many fields and has made major contributions to our understanding of biology and medicine. Translation of this technology and assays to patient testing has been limited despite grand expectations. The amyloid protein identification by LC–MS test is one successful example of the adaptation of this technology by molecular and clinical pathology laboratories. Through the illustration of this assay, we will address some of these challenges and outline a process for validation and implementation of mass spectrometry–based proteomics in the molecular pathology laboratory.

## Highlights

• Road map for validation & implementation of a clinical MS proteomics test.
• Amyloid subtyping is a successful implementation of proteomics in clinical testing
• A diagnostic that enables accurate classification & supports targeted treatment.
• Assay resulted in discovery of novel forms of amyloidosis.

## Graphical Abstract

**Figure:**
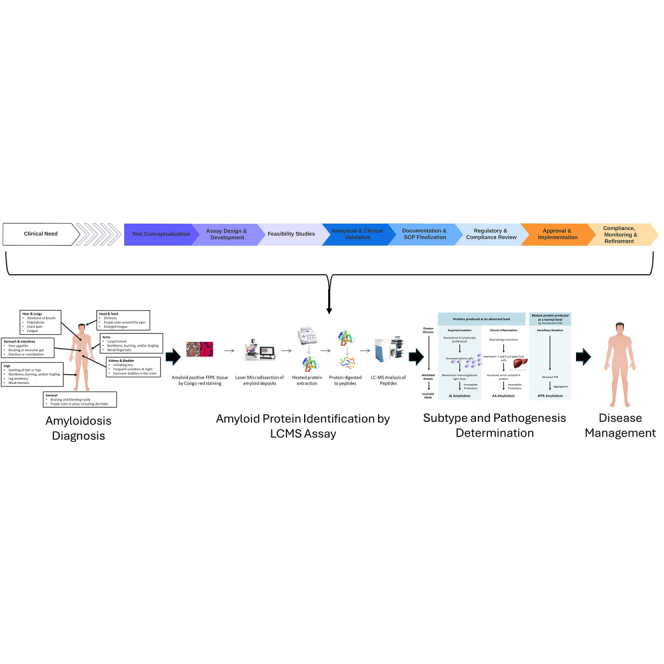

## In Brief

Amyloid protein identification by LC–MS test is a clinical pathology assay that has been validated and implemented for patient testing. A total of 223 clinical cases have been reported and provided disease classification information, allowing for proper disease management. This successful example of a mass spectrometry (MS)–based clinical test can serve as a case study for future molecular pathology assay development and validation.

Since its emergence as a high-resolution tool for comprehensive analysis of proteomes, MS-based proteomics has found widespread applications in basic science and clinical research with the promise of broad clinical translation. Yet, there has been limited success in the implementation of liquid chromatography, MS-based (LC–MS) proteomic assays in patient testing. There are several reasons for this failure, including infrastructure costs, lack of computational support, cultural elements, but perhaps most importantly, lack of know-how for the conceptual and regulatory implementation of promising ideas.

The most common route for LC–MS–based proteomics tests to be introduced into the clinical laboratory would be as a laboratory-developed test (LDT). An LDT is defined in the United States by the Food and Drug Administration as an *in vitro* diagnostic test that is “designed, manufactured, and used within a single laboratory that is certified under Clinical Laboratory Improvement Amendments of 1988 (CLIA) and meets the regulatory requirements under CLIA to perform high complexity testing” (1). CLIA is administered by the Centers for Medicare and Medicaid Services. In the United States, laboratories performing these high complexity tests are required to have and maintain CLIA certification by adhering to stringent requirements and encouraged to also follow standards of the International Organization for Standardization (ISO) (2, 3). ISO is a network of national bodies that develops a wide range of international standards for quality management systems, and ISO 15189 is a standard that specifies requirements for quality and competence in medical laboratories. Accreditation to ISO 15189 is not required in the United States but was a requirement for medical laboratories in 60 countries as of 2015 (2).

Both CLIA-licensed laboratories and ISO-accredited laboratories undergo regular inspections by accrediting organizations to ensure compliance. The College of American Pathologists is an example of an accrediting organization that is both approved by the Centers for Medicare and Medicaid Services and offers ISO accreditation (4). In addition to regulatory and required standards of CLIA and ISO 15189, the Clinical Laboratory Standards Institute is a nonprofit organization that rigorously develops and publishes consensus-based practical guidelines for many laboratory procedures (5). Clinical Laboratory Standards Institute standards and guidelines are helpful references for the implementation of regulatory requirements. In summary, LDTs are high complexity tests that require validation, rigorous quality control (QC), proficiency testing, and specially trained personnel to perform the assays. Since LC–MS–based testing is most likely to enter clinical patient testing as LDTs, these organizational relationships and processes are critical to understand prior to translation.

One of the few clinical laboratory applications of LC–MS–based proteomics is an LDT developed for the identification of the causative proteins in amyloid deposits (6). This test characterizes the tissue biopsy proteome for confirmation of amyloid deposition and for differentiation between the subtypes of this heterogeneous group of diseases. Determination of the causative protein(s) is critical to ensure proper treatment, as the subtypes differ in pathogenesis and clinical management.

All types of amyloidosis are characterized by the deposition of protein fibrils in extracellular regions, resulting in the displacement and damage of healthy tissue. There are approximately 30 known causative proteins (7). The most common subtypes of amyloidosis are amyloid light chain (AL), caused by immunoglobulin light chains originating from a neoplastic plasma cell population, and transthyretin amyloidosis (ATTR), caused by either wildtype or mutated transthyretin. These diseases lead to organ damage, dysfunction, and death, which is why diagnosis, characterization, and management of the diseases are critical (8).

Diagnosis of amyloidosis requires a biopsy of an affected tissue and staining of the tissue with Congo red dye. Congo red is bound specifically by amyloid fibrils, causing amyloid deposits to appear orange red in color under bright field microscopy. In addition, these regions produce a signature apple-green birefringence under polarized light. Following the identification of amyloid deposits in a tissue biopsy, the subtype of the disease must be determined by identifying the causative protein. Although immunohistochemistry (IHC) is the most widely available technique for identifying the causative protein, it is limited by the availability of antibodies, a lack of sensitivity, and crossreactivity in amyloid deposits (9). These limitations served as motivation for the development of the amyloid protein identification by LC–MS test in 2007 by Vrana *et al.* (6, 10).

Variations of this original assay have been adapted by reference laboratories, specialized disease centers, and academic centers worldwide (11, 12, 13, 14). It is currently the international gold standard for the classification of amyloidosis and has improved the patient diagnosis process and treatment outcomes. Knowledge of how to implement this assay is often shared between institutions informally, and dissemination of this information is important for successful test validation and implementation (15). Expertise in MS-based proteomics, pathology, and the disease area itself is critical, but without understanding of the validation and implementation requirements, transition of an assay to patient testing is difficult, even with strong collaborative teams.

**Fig. 1:**
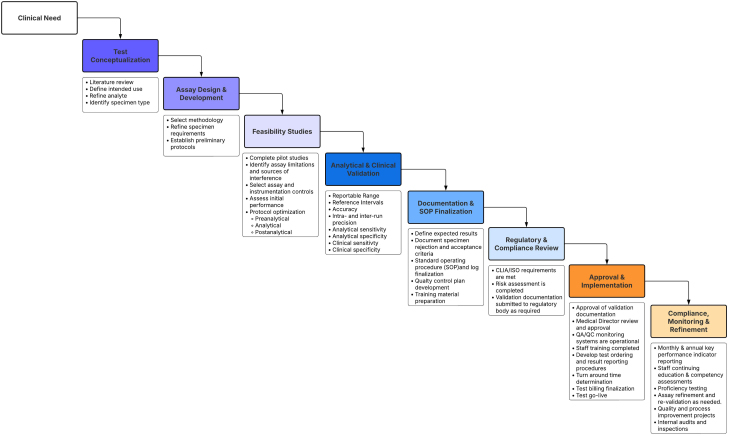
**Molecular pathology assay road map.** Molecular pathology proteomics assay development, validation, and implementation road map. An outline of the different phases of clinical test development and the specific tasks involved.

This document will discuss many of the conceptual, technical, quality management, and regulatory challenges through the example of the implementation of the amyloid protein identification by LC–MS assay at our institution, with the aim of broader clinical adaptation of this technology by molecular pathology laboratories. Figure 1 outlines components of the development, validation, implementation, and long-term compliance stages.

## Experimental Procedures

### Experimental Design and Statistical Rationale

Formalin-fixed paraffin-embedded (FFPE) tissue biopsies were identified by searching the pathology laboratory information system at Memorial Sloan Kettering Cancer Center for cases with a morphology-based amyloidosis diagnosis between 2011 and 2016. A set of 54 amyloidosis biopsy cases with previously determined subtypes were selected. Previous methodology for subtype determination included IHC, genetic testing for causative protein variants, and evaluation for plasma cell neoplasms, including serology workup and bone marrow biopsy.

Morphology and Congo red staining were reviewed with a pathologist to confirm the presence of amyloid deposits. Of the 54 cases, 40 were included in the validation, as the other 14 did not have sufficient tissue remaining on the FFPE tissue blocks. The sample size was limited based on the availability of cases with sufficient tissue. Amyloidosis is a rare disease, and a larger sample size was not possible. Tissue sections were cut 10 μm thick and placed on coated glass slides (DIRECTOR slides). The number of FFPE tissue sections required for each biopsy varied depending on the size of the biopsy and the extent of amyloid deposition. A minimum of 50,000 μm^2^ of Congo red–positive tissue is required per replicate. The number of tissue sections was determined during slide review with the pathologist.

**Fig. 2:**
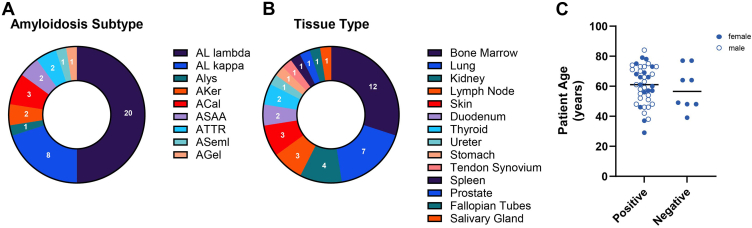
**Validation case statistics.** A selection of 40 amyloid cases were utilized for validation of the amyloid protein identification by LC–MS assay. *A,* this set of specimens represented nine different disease subtypes and (*B*) 14 tissue types. *C,* the patient cohort was representative of the general population of amyloidosis patients (male—*white circle* outlined by *blue* and female—*solid blue circles*).

The cases selected represented nine disease subtypes (Fig. 2*A*) and 14 tissue types (Fig. 2*B*). For negative samples, eight healthy myometrium tissue samples were formalin fixed and paraffin embedded. All negative controls came from patients without a history of amyloidosis and were reviewed with a pathologist to confirm that amyloid deposits were not present in the tissue. The amyloidosis patients were 29 to 84 years of age at the time of the biopsy, with an average age of 60 years (Fig. 2*C*). Negative myometrium tissue was collected from patients ranging from 39 to 77 years of age and an average of 58 years. Myometrium tissue and 16 of the amyloidosis biopsies were from female patients, with the remaining 24 amyloidosis tissue samples from male patients (Fig. 2*C*). No other demographic information was considered in the selection of cases.

All specimen slides were deidentified using a patient code starting with P1 for the first patient, P2 for the second case, and so on. A minimum of two and a maximum of three technical replicates were prepared for each case. Each replicate was assigned a letter A, B, or C. Each replicate contained 50,000 μm of laser microdissected tissue in a tube. All samples were prepared and analyzed by LC–MS between June and September 2016. Use of tissue specimen was approved by the Institutional Review Board of Memorial Sloan Kettering Cancer Center (Institutional Review Board no.: 16-1314) and abides by the Declaration of Helsinki principles.

### Amyloid Sample Analysis

#### FFPE Tissue Preparation

FFPE tissue sections were cut at 10 μm thick, placed onto DIRECTOR slides (Expression Pathology) and stained with Congo red dye. Congo red–positive tissue areas were identified by bright-field and fluorescence microscopy on a laser microdissected (LMD) 6500 microdissection microscope (Leica Microsystems). A total of 50,000 μm^2^ of Congo red–positive tissue was laser microdissected into the cap of a 0.5 ml microcentrifuge tube containing 50 μl of TEZ working buffer (10 mM Tris/1 mM EDTA/0.002% Zwittergent 3-16 [Calbiochem]) for each replicate. Tissue samples were heated at 90 °C for 30 min and an additional hour at 65 °C with shaking at 750 RPM on a thermomixer (Eppendorf), followed by 30 min of sonication in a water bath. Samples were digested overnight at 37 °C with 1 μg of trypsin (Promega). Then desalted using C18 spin columns (Harvard Apparatus) and dried in a SpeedVac concentrator. The resulting peptide samples were reconstituted in 20 μl 0.1% formic acid, and 4.5 μl were transferred to an autosampler vial.

#### LC-MS Analysis

LC–MS analysis of the digested tissue samples was performed on an UltiMate 3000 RSLCnano and Q Exactive Plus mass spectrometer (Thermo Fisher Scientific). A 4 μl aliquot (1/5 total sample) of each peptide sample was loaded onto an Acclaim PepMap 100 C18 trap column in line with a 50 cm EASY Spray column. Peptide samples were separated using a 40-min gradient from 3.8% to 25.8% mobile phase B followed by an additional 10-min gradient to 37.6% mobile phase B. Mobile phase B was 0.1% formic acid in acetonitrile, and mobile phase A was 0.1% formic acid in water. Data were acquired using a top 10 data-dependent acquisition (DDA) method with a resolution of 70,000, 1e6 automatic gain control (AGC) target, and maximum injection time (IT) of 50 ms for the full scan. The MS2 spectra were collected at 17,500 resolution, with an AGC target of 1e5 and maximum IT of 110 ms. Isolation window was set to 1.5 *m/z*, normalized collision energy (NCE) of 25, and dynamic exclusion was 20 s. For each patient specimen, a blank was run prior to the samples using the same LC–MS method as the amyloid samples but injecting 2 μl of mobile phase A.

#### Data Analysis

Amyloidosis sample and presample blank raw files were searched using Byonic (Protein Metrics LLC, version 2.11.0) within Proteome Discoverer 2.1 (Thermo Fisher Scientific) against the UniProt human reference database (downloaded July 25, 2016, with 20,197 entries) with the manual addition of immunoglobulin variant domains and common contaminant protein sequences. Precursor mass error tolerance was set to 10 ppm, and fragment mass error tolerance was 0.02 Da. Trypsin fully specific cleavage was selected with two missed cleavages allowed. Modifications allowed are specified in Supplemental Table S1, and a maximum of two common and one rare variable modification were allowed per peptide. The protein false discovery rate (FDR) was set to 1% for the search. Only high-confidence peptide spectral matches (PSMs) with an FDR of 1% or less and a Byonic score of 300 or higher were considered in the analysis. Proteins with at least one unique peptide were considered for interpretation. An average PSM value and % CV were calculated for amyloid biomarker proteins and subtype proteins to ensure the variation was less than 20% between replicates. Following the search of the individual files, a multiconsensus file was created in Proteome Discoverer, which combines the results into one file, allowing for review of the blank and patient specimens replicate results side by side.

#### Data Interpretation

Blank and patient specimen search results were evaluated to determine the total number of protein groups identified and the relative abundance of detected proteins based on the number of PSMs. A PSM is an instance where a peptide sequence has been identified for that protein and includes redundantly identified peptides. For patient samples, at least two of three replicates had to be concordant and if fewer than three replicates, all must be concordant. All patient specimens required a minimum of 50 protein groups and less than 30% missed cleavage across all identified proteins. Protein groups contain proteins that cannot be unambiguously identified by unique peptides in the search output.

For a specimen to be positive for amyloid, apolipoprotein E, and serum amyloid P, which are general protein biomarkers of amyloid deposits, had to be identified with five or more PSMs. The amyloid disease type could only be determined if the subtype protein(s) were identified with five or more PSMs, and all other known subtype proteins were not detected or present with less than five PSMs. For example, if greater than five PSMs for both APOE and SAMP were identified, transthyretin was present with 50 PSMs, and no other known subtype protein was identified in that case, then it would be interpreted as ATTR. If in the same example, immunoglobulin light chain kappa was also present, but with three PSMs, it would still be ATTR.

A case was determined to be inconclusive for subtype if results met APOE and SAMP requirements, but a subtype could not be conclusively determined. A specimen was called negative if there were fewer than five PSMs for APOE and SAMP. This would apply to negative controls or false-positive cases if there was nonspecific Congo red staining. No false-positive Congo red cases were included in the validation cohort. Example results for an AL and an ATTR case are shown in Supplemental Figure S3. PSM cutoffs were determined by pilot studies (data not shown).

Blanks run prior to specimen analysis were used to determine if there was carryover from previous analyses. Blank run search result requirements were set to less than 50 protein groups and less than five PSMs for APOE, SAMP, and for all known amyloid subtype proteins. If there was evidence of carryover in the blank, then the patient samples were reanalyzed by LC–MS.

### Precision Study

For the amyloid protein identification by LC–MS test, intrarun and inter-run precision testing of three specimens (P18, P28, and P48) was performed. Due to sample volume limitations, the replicates for each sample were pooled to generate a single sample for each specimen that could be injected multiple times. Pooled samples were stored at 4 °C in the autosampler tray for 7 days and analyzed twice on days 1, 3, 5, and 7 for a total of eight injections per specimen. Results were evaluated based on PSMs as previously described and compared for concordance.

### Instrument Precision and QC Assessment

#### Daily Instrument Evaluation

LC–MS instrument precision was evaluated by determining the stability of retention times, peak widths, MS1 peak intensity, and mass accuracy of precursor ions in the MS1 spectra of five peptides in the Promega 6 × 5 LC–MS/MS reference. Promega 6 × 5 LC–MS/MS reference material consists of six peptides, each with five isotopologs. The reference mix was prepared to a concentration of 100 fmol/μl, transferred to an autosampler vial, and stored in the autosampler tray at 4 °C for 14 days. A microliter of the standard was loaded onto a 50 cm EASY Spray column and separated using the same method as the amyloid specimen. Data were acquired using a top 10 DDA method as described above for the acquisition of amyloid samples. Data were analyzed with the PReMiS software produced by Promega Corporation.

To determine intraday instrument precision, 100 fmol of the Promega 6 × 5 LC–MS/MS reference material was analyzed as outlined above, nine times across a 24-h period. Peptide retention time in minutes, precursor ion mass accuracy (ppm), precursor peak intensity, and precursor peak width in seconds were recorded for each run. For each of the five peptides, the average retention time, precursor mass accuracy, and precursor peak width values were calculated along with standard deviation and percent variance (%CV). For interday precision, the same vial of Promega 6 × 5 LC–MS/MS reference material was injected three times on days 1, 3, 5, 7, and 14. The daily average was calculated as well as the average across days 1 to 7 and days 1 to 14. Percent variance was calculated for days 1 to 7 and days 1 to 14 for retention time, precursor peak intensity, and precursor peak width with a %CV cutoff of 20% or less. A cutoff of ±3 ppm for mass accuracy was set for both intraday and interday precision.

Promega 6 × 5 LC–MS/MS was replaced with Pierce peptide retention time calibration mixture (PRTC) during assay implementation. A microliter of a 100 fmol/μl solution of PRTC was loaded as previously described. Data were acquired using a parallel reaction monitoring (PRM) method, including an MS1 full scan followed by five PRM MS2 scans. The full scan spectrum was acquired with a resolution of 70,000, 1e6 AGC target, and a maximum IT of 50 ms. The MS2 spectra were collected at 17,500 resolution, with an AGC target of 2e5 and maximum IT of 110 ms. We utilized an isolation window of 1.5 *m/z* and NCE of 25. PRM target list contained the 15 PRTC peptides.

Intraday and interday evaluations were performed to benchmark the standard. Data were analyzed in Skyline to determine mass accuracy, peak width, peak intensity, and retention time stability (16). The peptide precursor and the top three most abundant transitions (fragment ions) are monitored in the Skyline document. MS1 and MS2 mass accuracy must be ±3 ppm of the theoretical masses, or mass calibration was required. The full width at half maximum of the peaks must be 0.25 min or less. Peak intensity minimums were set for each peptide based on cumulative data from analyses over the course of 3 months. Retention times are recalibrated whenever the mobile phase is replaced or a new column is installed. A minimum of five sequential runs are analyzed to calculate the average retention time in minutes. Then the retention times must be ±1.5 min of the average value. A dot product value of 0.97 or greater is required when comparing the relative abundance of fragment ions in the MS2 spectra to an in-house built spectral library.

#### Weekly Instrument Evaluations

HeLa protein digest standard (Thermo Scientific) was prepared at 100 ng/μl, transferred to an autosampler vial, and stored in the autosampler tray at 4 °C for 14 days. On day 1, five replicate analyses were performed as described below. In addition, on days 3, 5, 7, and 14, a single analysis was performed. Two microliters of the standard were loaded onto a 50 cm EASY Spray column and separated using a 70-min gradient from 4.5% to 27% mobile phase B, followed by an additional 7 min to 40.5% mobile phase B. Data were acquired using a top 10 DDA method. The full scan spectrum was acquired with a resolution of 70,000, 1e6 AGC target, and a maximum IT of 30 ms. The MS2 spectra were collected at 17,500 resolution, with an AGC target of 1e5 and a maximum IT of 200 ms. We utilized an isolation window of 1.5 *m/z*, NCE of 25, and set dynamic exclusion to 20 s.

HeLa raw files were searched using Byonic (version 2.11.0) within Proteome Discoverer 2.1 (Thermo Fisher Scientific) against the UniProt human reference database as mentioned above. Precursor mass error tolerance was set to 10 ppm, and fragment mass error tolerance was 0.02 Da. Trypsin fully specific cleavage was selected with two missed cleavages allowed. Carbamidomethylation on cysteines was a static modification, whereas oxidation on methionine and deamidation on asparagine and glutamine were set as common variable modifications with a maximum of two common variable modifications per peptide. Protein FDR was set to 1% for the search. Only high-confidence PSMs with an FDR of 1% or less and a Byonic score of 300 or higher were considered in the analysis. Proteins with at least two unique peptides were considered for interpretation. Protein group, peptide group, PSMs, and the number of MS2 spectra were recorded. The percentage of MS/MS converted to PSMs was calculated, and the base peak chromatogram intensity was also recorded. Variance across the multiple injections on day 1 and across days 1 to 14 was calculated and used to set minimum expected values for protein groups identified, percent MS/MS conversion to PSM, and the base peak chromatogram intensity.

The HeLa lysate LC–MS method was also modified to align with recommendations from the instrument vendor. HeLa lysate digest was separated using a longer 100-min gradient from 3.8% to 19.6% mobile phase B, followed by an additional 20 min to 30.2% mobile phase B (mobile phases previously described). Data were acquired using a top 10 DDA method with one targeted single ion monitoring (tSIM) scan. The full scan spectrum was acquired with a resolution of 70,000, 3e6 AGC target, and a maximum IT of 60 ms. The MS2 spectra were collected at 17,500 resolution, with an AGC target of 1e5 and maximum IT of 60 ms. We utilized an isolation window of 2 *m/z*, NCE of 25, and set dynamic exclusion to 20 s. The tSIM scan was acquired at 35,000 resolution, 5e4 AGC target, 60 ms maximum IT, and a 2.0 *m/z* isolation window. The inclusion list for the tSIM scan contained the 15 PRTC peptides. Data were searched using Byonic within Proteome Discoverer as described above. In addition to reassessing intraday and interday variability, minimum results were established so that protein groups identified must be greater than 1800, the percent of MS/MS converted to PSMs is greater than 45%, and the base peak intensity is greater than or equal to 5e8 AUC. HeLa digest lysate was analyzed following weekly maintenance, any other instrument repairs, or service, in addition to prebatch and postbatch. PRTC was analyzed at least once every 24 h in addition to prebatch and postbatch. If any of these results do not meet requirements, the clinical specimen cannot be analyzed, and troubleshooting begins.

### Assay Optimization

#### Reduction and Alkylation of Amyloidosis Samples

Three patient biopsies that were previously analyzed were selected. Four replicate samples were microdissected from two of the specimens and two replicates from the third specimen because of limited tissue. All samples were prepared as previously described, with the addition of reduction and alkylation steps to two of the samples per patient specimen prior to digestion with trypsin. DTT was added at a final concentration of 0.8 mM, and samples were heated at 57 °C for 45 min, cooled to room temperature, and iodoacetamide was added at a final concentration of 4 mM and allowed to incubate at room temperature for 60 min. All samples were digested with trypsin, and peptides were desalted as described above. Aliquots of the peptide samples were analyzed by LC–MS as previously outlined. Data were analyzed using Byonic within Proteome Discoverer using the same workflow as described previously, with the addition of carbamidomethylation of cysteine to the static modification list. Data were analyzed to determine the sequence coverage of proteins of interest.

#### Microscope Slide Evaluation

FFPE biopsy sections, 10 μm thick, were cut from a previously characterized shoulder biopsy with AL lambda amyloid deposition. Sections were mounted onto DIRECTOR, PEN membrane (Leica Microsystems), charged glass slides, or uncharged glass slides and stained with Congo red. Congo red–positive tissue areas were identified by bright-field and fluorescence microscopy, and a total of 50,000 μm^2^ of Congo red–positive tissue was microdissected into 0.5 ml microcentrifuge tube caps containing 50 μl of TEZ working buffer as previously described. All sample preparation steps were followed as outlined above, and results were compared to assess the compatibility of other slide types with the assay.

#### DIRECTOR Slide *Versus* PEN Membrane Slide

FFPE biopsy sections, 10 μm thick, were cut from three previously characterized amyloid biopsies. The biopsies had previously been characterized; one was ATTR and the other two were AL lambda. Sections were mounted onto the DIRECTOR and PEN membrane slides for all three biopsies. Congo red–positive tissue (50,000 μm^2^) was microdissected into 0.5 ml microcentrifuge tube caps containing 50 μl of TEZ working buffer as previously described. All sample preparation steps were followed as outlined above, and results were compared between the two slide types.

## Results

### Amyloid Protein Identification by LC–MS Validation

#### Accuracy

To assess accuracy, the amyloidosis subtype determined previously through established methods, including IHC, was compared with the subtype determined by the LC–MS assay. Results were reported as concordant or nonconcordant for subtype (Table 1). All amyloidosis-positive cases were found to be positive, and all negative specimens were negative based on the criteria previously outlined. Disease subtype determination was concordant in all 40 positive cases. In addition, material from 12 cases was sent to another institution with an MS-based assay approved for clinical use by the same regulatory body (NYS Department of Health), and those results were also concordant for subtype.

**Table 1:** Amyloid protein identification by LC–MS test was validated using 40 well-characterized patient samples and 8 negative controls

Specimen no.	Patient identifier	Sex	Age	Tissue type	Average LMD tissue (μm^2^)	Average no.: of proteins identified LC–MS	Gold standard diagnosis	Amyloid protein identification by LC–MS results			LC–MS diagnosis	Concordance
								APOE	SAP	Subtype protein(s)		
1	P1	F	56	Fallopian tubes & ovaries	66,487	242	AL lambda	✓	✓	Ig lambda-2 C region	AL lambda	Yes
2	P2	M	66	Lung	65,253	325	AL lambda	✓	✓	Ig lambda-2 C region	AL lambda	Yes
3	P3	M	75	Lung	66,391	322	AL lambda	✓	✓	Ig lambda-2 C region and V region 4A	AL lambda	Yes
4	P4	F	66	Lung	67,650	306	AL lambda	✓	✓	Ig lambda-2 C region	AL lambda	Yes
5	P5	M	48	Lymph node	66,397	367	AL lambda	✓	✓	Ig lambda-2 C region	AL lambda	Yes
6	P6	M	46	Bone marrow	66,932	377	AL lambda	✓	✓	Ig lambda-2 C region and V1 region New	AL lambda	Yes
7	P7	F	61	Bone marrow	68,703	251	AL lambda	✓	✓	Ig lambda-2 C region and V region 4A	AL lambda	Yes
8	P8	M	61	Bone marrow	63,076	455	AL kappa	✓	✓	Ig kappa C region and V-I region WAT/Mev	AL kappa	Yes
9	P9	F	57	Bone marrow	67,694	346	AL lambda	✓	✓	Ig lambda-2 C region and V-II region Hil/Bau	AL lambda	Yes
10	P10	F	60	Bone marrow	68,252	395	AL lambda	✓	✓	Ig lambda-2 C region	AL lambda	Yes
11	P11	M	38	Lung	67,188	377	AL lambda∗∗	✓	✓	Ig lambda-2 C region	AL lambda	Yes
12	P12	M	46	Lymph node	63,865	379	ALys∗∗	✓	✓	Lysozyme C	ALys	Yes
13	P13	M	73	Kidney	67,535	442	AL kappa∗∗	✓	✓	Ig kappa C region and V-I region Wes	AL kappa	Yes
14	P16	M	54	Lung	67,785	399	AL kappa	✓	✓	Ig kappa C region and V-III region SIE/IARC	AL kappa	Yes
15	P17	M	51	Skin	65,081	209	Cutaneous keratin-associated amyloidosis	✓	✓	Keratin, type I, 14 and keratin type II, 5	Keratin-14/5	Yes
16	P18	M	71	Skin	66,605	221	Dermal amyloidosis	✓	✓	Keratin, type I, 14 and keratin type II, 5	Keratin-14/5	Yes
17	P21	M	55	Lung	67,162	361	AL kappa∗∗	✓	✓	Ig kappa C region	AL kappa	Yes
18	P22	M	52	Bone marrow	66,394	291	AL kappa	✓	✓	Ig kappa C region and V-I region Scw/Mev	AL kappa	Yes
19	P23	F	29	LN, thyroid	67,634	272	ACal	✓	✓	Calcitonin and Calcitonin gene-related pep 2	ACal	Yes
20	P25	F	66	Thyroid lobe	66,844	396	ACal	✓	✓	Calcitonin and Calcitonin gene-related pep 2	ACal	Yes
21	P26	F	68	Thyroid	66,612	301	ACal	✓	✓	Calcitonin and Calcitonin gene-related pep 2	ACal	Yes
22	P27	F	69	Ureter	65,958	263	AL lambda	✓	✓	Ig lambda-2 C region and V-III region LOI	AL lambda	Yes
23	P28	M	58	Stomach	60,272	632	AL lambda	✓	✓	Ig lambda-1 C region and V-II region Win	AL lambda	Yes
24	P29	F	73	Bone marrow	66,354	246	AL lambda	✓	✓	Ig lambda-2/3 C region and V-III region LOI	AL lambda	Yes
25	P30	M	73	Kidney	63,251	331	AL lambda	✓	✓	Ig lambda-2/3/7 C region	AL lambda	Yes
26	P39	F	79	Kidney	65,147	240	ASAA∗∗	✓	✓	Serum amyloid A-1 and A-2	ASAA	Yes
27	P40	M	74	Synovial tendon	67,172	287	ATTR∗∗	✓	✓	Transthyretin	ATTR	Yes
28	P41	F	46	Spleen	66,461	415	AL lambda∗∗	✓	✓	Ig lambda-2 C region	AL lambda	Yes
29	P42	F	57	Skin	66,333	263	AL lambda∗∗	✓	✓	Ig lambda-2 C region and V-III region LOI	AL lambda	Yes
30	P43	M	59	Bone marrow	64,401	247	AL kappa	✓	✓	Ig kappa C region and V-IV region Len	AL kappa	Yes
31	P44	F	37	Lung	66,783	299	AL lambda∗∗	✓	✓	Ig lambda-2 C region	AL lambda	Yes
32	P45	M	42	Bone marrow	67,340	287	AL lambda∗∗	✓	✓	Ig lambda-2 C region and V-VI region AR	AL lambda	Yes
33	P47	M	63	Prostate	67,995	309	ASemI∗∗	✓	✓	Semenogelin-I	ASemI	Yes
34	P48	M	70	Kidney	58,897	235	ASAA	✓	✓	Serum amyloid A-1	ASAA	Yes
35	P49	M	73	Gastrointestinal tract	57,317	253	ATTR∗∗	✓	✓	Transthyretin	ATTR	Yes
36	P50	F	78	Salivary gland	66,398	253	AGel	✓	✓	Gelsolin	AGel	Yes
37	P51	M	48	Gastrointestinal tract	66,131	552	AL lambda	✓	✓	Ig lambda-2 C region	AL lambda	Yes
38	P52	M	68	Bone marrow	66,246	350	AL kappa	✓	✓	Ig kappa C region and V-I region Mev/Rei	AL kappa	Yes
39	P53	M	84	Bone marrow	67,542	378	AL kappa	✓	✓	Ig kappa C region	AL kappa	Yes
40	P54	F	75	Bone marrow	66,696	318	AL lambda	✓	✓	Ig lambda-2 C region	AL lambda	Yes
41	P55	F	77	Uterus	68,221	211	No amyloidosis	-	-	-	Negative	Yes
42	P56	F	77	Uterus	69,864	270	No amyloidosis	-	-	-	Negative	Yes
43	P57	F	48	Uterus	68,472	313	No amyloidosis	-	-	-	Negative	Yes
44	P58	F	39	Uterus	71,627	321	No amyloidosis	-	-	-	Negative	Yes
45	P59	F	49	Uterus	64,087	246	No amyloidosis	-	-	-	Negative	Yes
46	P60	F	64	Uterus	65,373	257	No amyloidosis	-	-	-	Negative	Yes
47	P67	F	48	Uterus	61,860	242	No amyloidosis	-	-	-	Negative	Yes
48	P68	F	64	Uterus	64,805	225	No amyloidosis	-	-	-	Negative	Yes

All cases were found to meet requirements, and subtype was concordant with the classification determined by previous gold standard methods. Cases with ∗∗ after the subtype were also tested at another institution with a comparable mass spectrometry-based assay and results were found to be concordant.

#### Precision

For the amyloid protein identification by LC–MS test, intrarun and inter-run precision testing used three specimens (P18, P28, and P48). Results from the eight injections of each sample were evaluated, interpreted, and compared for concordance (Table 2). The duplicate injections on the same days were to assess intrarun precision, and inter-run precision was assessed by comparing the results across the days. The % CV for PSMs for APOE and SAMP was 20% or less within a day and across days. Subtype biomarker(s) PSMs %CV were also 20% or less intraday and interday. All cases met requirements to be called positive for amyloidosis, and subtype determination was concordant across all injections and with the original LC–MS analysis of that specimen during the validation cohort analysis.

**Table 2:** Precision and specimen stability was determined through interday and intraday analysis of three patient samples

P18	No. of PSM amyloid biomarkers		No. of PSM subtype biomarkers	
Day	Apolipoprotein E	Serum amyloid P	Keratin type II-14	Keratin type I-5
1	10	13	15	14
3	11	12	16	20
5	9	15	17	16
7	12	13	15	16
% CV	10.7	10.9	6.7	15.2

P28	No. of PSM amyloid biomarkers		No. of PSM subtype biomarkers
Day	Apolipoprotein E	Serum amyloid P	Ig lambda light chain
1	11	19	20
3	14	26	19
5	12	29	22
7	12	29	20
% CV	10.3	17.6	6.4

P48	No. of PSM amyloid biomarkers		No. of PSM subtype biomarkers
Day	Apolipoprotein E	Serum amyloid P	Serum amyloid A-1
1	6	7	19
3	7	6	20
5	7	5	20
7	6	5	22
% CV	7.5	19.7	6.5

Patient 18 was an Aker subtype caused by keratin deposition; patient 28 was an AL lambda subtype case, and patient 48 was ASAA caused by serum amyloid A protein. PSM values were less than 20% across interday and intraday testing for APOE, SAMP, and subtype biomarkers.

#### Analytical Sensitivity and Specificity

The lower end of the analytical sensitivity of the assay is set at 50,000 μm^2^ of tissue. All LMD-prepared samples in the validation cohort contained a minimum of 50,000 μm^2^, meaning that analytical sensitivity was 100% based on the established guidelines of the assay.

Analytical specificity is the assay's ability to detect the analyte(s) and differentiate them from other molecules in the specimen. Analytical specificity was assessed by the detection of the general amyloidosis biomarkers, APOE and SAMP, in all samples and the lack of these proteins in the negative controls. These analytes were detected in all amyloidosis samples and in none of the negative control samples. In addition, the detection of subtype-specific proteins in each sample and the lack of all subtype proteins in the negative controls confirmed the analytical specificity of the test.

#### Clinical Sensitivity

Clinical sensitivity is the ability of the test to identify the presence of a disease, illness, or abnormality correctly. All 40 amyloidosis cases analyzed in the validation had established diagnosis based on gold standard practices as previously described. All 40 amyloidosis cases were positive based on the established criteria, and subtype determination was concordant with the existing diagnosis. Clinical sensitivity was 100% in the validation of the MS-based assay (Table 1).

#### Clinical Specificity

Clinical specificity is the ability of the assay to identify the absence of a disease or illness correctly. Clinical specificity was also 100% as all eight negative specimens were correctly determined to be negative for amyloidosis. There were no false-positive results.

#### Sample Stability

Specimen stability was evaluated in the precision testing as the samples were kept in the refrigerated autosampler for a week. Results were concordant from day 1 through day 7, indicating the samples could be stable for at least 7 days when stored at 4 °C (Table 2). In addition, a subset of specimens were stored in a −80 °C freezer following initial validation analysis. These samples were thawed at 1 week and again at 1 month for follow-up LC–MS analysis. The results were concordant for all analyses and ensured that prepared samples were stable in case of instrument downtime or reanalysis requirements (data not shown).

### Instrument Precision and QC Assessment

#### Instrument Precision Evaluations

The Promega 6 × 5 peptide reference mix was analyzed nine times in a single day to assess intraday precision and then four times a day over the course of 14 days on days 1, 3, 5, 7, and 14. Retention time variability was less than 1% CV, and peak width variability was less than 10% CV (Table 3). Peak height had a CV of under 20%, except for the most hydrophobic peptide, which had greater variability in peak intensity. Due to the high variation associated with peptides that elute at the farther ends of a gradient, the peak intensity values of the last eluting peptide were not used as a rejection criterion for the peptide standard. Mass accuracy was within ±3 ppm from the theoretical values across all days. The mass accuracy did start to drift closer to −3 ppm on day 14 (Table 4). The Q Exactive Plus mass spectrometer can achieve mass accuracy of less than 1 ppm, and all data analyses are set to expect a mass accuracy of less than or equal to 10 ppm; therefore, the goal is to maintain mass accuracy at ±3 ppm during normal operation (17). To maintain that level of performance, a weekly instrument evaluation and mass calibration are performed.

**Table 3:** Instrument precision and stability were assessed with an LC–MS retention time peptide standard

Replicate	1	2	3	4	5	6	7	8	9	% CV
Peptide sequence	Retention time (min)									
LASVSVSR	26.9	27.0	26.9	27.2	27.2	27.1	27.0	27.0	27.1	0.29
YVYVADVAAK	41.8	41.9	41.8	42.1	42.2	42.0	41.9	42.0	41.9	0.28
VVGGLVALR	47.9	48.0	48.0	48.2	48.2	48.0	47.9	48.0	48.0	0.23
LLSLGAGEFK	57.1	57.2	57.3	57.4	57.4	57.3	57.2	57.3	57.3	0.14
LGFTDLFSK	76.6	75.6	76.7	76.7	76.7	76.6	76.5	76.6	76.6	0.09

Replicate	1	2	3	4	5	6	7	8	9	<3 ppm
Peptide sequence	Precursor mass accuracy (ppm)									
LASVSVSR	−1.17	−1.10	−1.45	−1.17	−1.38	−1.38	−1.38	−0.95	−0.88	Yes
YVYVADVAAK	−0.69	−1.12	−1.34	−0.91	−1.12	−1.34	−0.80	−1.02	−0.37	Yes
VVGGLVALR	−1.01	−1.68	−1.74	−1.61	−1.34	−1.48	−1.48	−1.08	−1.14	Yes
LLSLGAGEFK	−0.60	−1.17	−1.40	−0.94	−1.17	−1.40	−0.83	−0.83	−1.06	Yes
LGFTDLFSK	−0.80	−1.60	−1.26	−1.37	−1.15	−0.80	−0.92	−1.26	−1.15	Yes

Replicate	1	2	3	4	5	6	7	8	9	% CV
Peptide sequence	Precursor peak intensity (AU)									
LASVSVSR	3.5E8	3.5E8	3.6E8	3.8E8	3.7E8	3.7E8	3.6E8	3.7E8	3.6E8	2.83
YVYVADVAAK	3.0E8	2.9E8	2.6E8	2.8E8	3.0E8	2.9E8	2.9E8	2.8E8	2.8E8	4.76
VVGGLVALR	3.4E8	3.2E8	3.2E8	3.1E8	2.9E8	3.1E8	2.9E8	2.8E8	2.7E8	7.41
LLSLGAGEFK	2.6E8	2.3E8	2.3E8	2.2E8	2.1E8	1.9E8	1.9E8	1.8E8	1.7E8	14.51
LGFTDLFSK	1.3E8	1.3E8	1.2E8	1.1E8	1.0E8	9.5E7	9.5E7	8.0E7	7.3E7	20.62

Replicate	1	2	3	4	5	6	7	8	9	% CV
Peptide sequence	Precursor peak width (s)									
LASVSVSR	9.6	10.1	9.7	9.5	9.2	9.4	9.7	9.4	9.2	3.12
YVYVADVAAK	12.5	12.9	13.9	13.2	12.7	12.7	13.4	12.9	13.3	3.34
VVGGLVALR	13.0	14.0	12.6	14.5	14.1	13.5	12.9	13.4	12.9	4.82
LLSLGAGEFK	12.1	13.4	13.9	13.5	13.3	13.3	13.7	13.5	13.3	1.91
LGFTDLFSK	12.9	13.2	13.3	13.1	13.9	13.7	13.6	13.7	13.7	2.45

Intraday precision was measured with nine injections over a 24-h period. Metrics evaluated were retention time, precursor mass accuracy, precursor peak intensity, and precursor peak width.

**Table 4:** Instrument precision and stability were assessed with an LC–MS retention time peptide standard

Day	1	3	5	7	14	% CV (day 1–7)	% CV (day 1–14)
Peptide sequence	Average retention time (min)						
LASVSVSR	28.6	28.6	28.6	28.6	29.3	0.05	1.02
YVYVADVAAK	43.1	43.0	43.1	43.1	44.1	0.18	1.11
VVGGLVALR	49.2	49.1	49.3	49.3	50.3	0.17	1.00
LLSLGAGEFK	58.5	58.4	58.6	58.6	59.7	0.17	0.87
LGFTDLFSK	78.3	78.2	78.4	78.3	79.5	0.09	0.67

Day	1	3	5	7	14	<3 ppm
Peptide sequence	Mass accuracy					
LASVSVSR	−0.15	−0.54	−0.99	−0.99	−1.83	Yes
YVYVADVAAK	−0.29	−0.96	−1.12	−1.12	−1.99	Yes
VVGGLVALR	−0.55	−1.36	−1.54	−1.74	−2.49	Yes
LLSLGAGEFK	−0.43	−0.89	−1.20	−1.24	−2.13	Yes
LGFTDLFSK	−0.15	−0.49	−0.69	−0.97	−2.06	Yes

Day	1	3	5	7	14	% CV (day 1–7)	% CV (day 1–14)
Peptide sequence	Average peak intensity						
LASVSVSR	2.0E8	2.4E8	2.4E8	2.4E8	2.1E8	8.70	8.63
YVYVADVAAK	2.0E8	2.4E8	2.4E8	2.4E8	2.2E8	8.70	7.85
VVGGLVALR	3.0E8	3.0E8	2.9E8	2.7E8	2.3E8	4.88	10.61
LLSLGAGEFK	2.0E8	1.8E8	1.6E8	1.6E8	1.3E8	10.94	15.71
LGFTDLFSK	5.0E7	4.0E7	2.6E7	1.5E7	1.1E7	46.90∗	57.91∗

Day	1	3	5	7	14	% CV (day 1–7)	% CV (day 1–14)
Peptide sequence	Peak width (s)						
LASVSVSR	8.0	8.5	8.5	8.0	8.5	3.51	3.32
YVYVADVAAK	11.7	11.8	11.6	12.2	11.7	2.10	1.95
VVGGLVALR	13.0	12.6	13.1	12.3	12.4	2.68	2.69
LLSLGAGEFK	13.3	12.7	12.5	12.2	11.1	3.58	6.48
LGFTDLFSK	13.1	13.3	13.8	13.4	11.7	2.13	6.21

Interday precision was measured with three injections each day on days 1, 3, 5, 7, and 14. Metrics evaluated were retention time, precursor mass accuracy, precursor peak intensity, and precursor peak width.

∗ Peptide LGFTDLFSK elutes late and is not as reproducible as the other peptides due to its hydrophobicity and therefore the % CV is greater than 20 %.

As discussed, prior to the implementation of the assay, the peptide standard utilized in the laboratory was changed from the Promega 6 × 5 sample to the Pierce PRTC standard. The same intraday and interday analysis was performed using the PRTC standard analyzed *via* a PRM method. The open-source software Skyline (16) is used for analysis of PRTC data, which allows for evaluation and monitoring of additional key performance indicators, including fragment ion mass accuracy and MS2 spectra precision through dot product values. This simple yet complete system evaluation is performed at least once every 24 h to ensure instrument performance is optimal. Instrument downtime and patient result delays are minimized by early detection of instrument performance problems. PRTC is also evaluated at the beginning and end of each patient batch. A monthly Levey–Jennings chart of the PRTC peptide intensities provides a longitudinal review of instrument performance.

### Assay Optimization Postimplementation

#### Reduction and Alkylation of Amyloidosis Samples

Three specimens from patients with AL amyloidosis that were previously analyzed as part of the validation were used to evaluate the addition of reduction and alkylation steps into the sample preparation workflow. The number of PSMs identified for all proteins, including immunoglobulin light chains, did not change significantly (Supplemental Table S2). Sequence coverage of APOE and SAMP did not change with the addition of reduction and alkylation, but sequence coverage for immunoglobulin light chains increased by approximately 40% in all patient samples (Supplemental Table S3). Reduction and alkylation improved sequenced coverage for immunoglobulin light chains and were implemented into the assay workflow.

#### Microscope Slide Evaluation

Validation studies were performed using tissue mounted on DIRECTOR microscope slides for LMD. However, the DIRECTOR slides were often out of stock with long backorders. Alternative slide types were evaluated by cutting the same lung biopsy tissue onto DIRECTOR, PEN membrane, charged, and uncharged microscope slides. Performance was evaluated by comparing the number of protein groups identified overall and the number of PSMs for APOE, SAMP, and immunoglobulin lambda light chain. Sample microdissected from the DIRECTOR and PEN membrane slides resulted in similar values (Supplemental Table S4). The samples collected from the charged and uncharged glass slides did not meet requirements for amyloid-positive samples, despite the same area of tissue being collected from the same tissue sample.

#### DIRECTOR Slide *Versus* PEN Membrane Slide

The LMD specimens collected from both the DIRECTOR and PEN membrane slides were positive for amyloidosis based on the amyloid LC–MS assay requirements. All samples also met the requirements for subtype determination, and results were concordant with previous analysis and between the two slide types (Supplemental Table S5).

## Discussion

Variations of the LC–MS proteomics assay for amyloid subtype determination have proven very powerful, and this technology is uniquely suited to addressing this clinical question. This has been a prime example of when an LDT is critical, as existing technology of IHC was unable to provide definitive answers in many cases. LC–MS analysis does not require prior knowledge of disease subtypes, so new subtypes can also be discovered or confirmed using this technique. Some antibodies for subtype proteins, including those against kappa and lambda immunoglobulin light chains, produce high background staining. In addition, IHC assays do not exist for all relevant subtype proteins. This is an example of how LC–MS–based proteomics was a powerful replacement of an existing technology. Before discussing the specific validation results and implementation of this assay, it is useful to review some of the steps from conceptualization to validation as outlined in Figure 1.

### Design and Development

#### Specimen Selection

The choice of specimens used for a clinical LC–MS–based assay will depend upon the biomarker(s) being evaluated, where and how it is expressed, stability of the analyte, and other factors. Specimen collection container and handling requirements are critical to ensure all samples are comparable to one another and compatible with the downstream processing. This is especially important when considering LC–MS assays, as some reagents commonly used in clinical specimen collection vessels can introduce interfering compounds. Also, protease digestion or other degradation can occur if specimens are not handled properly, which will cause additional variation during the validation process. Deviations from the defined processing workflows may impact specimen stability and will be assessed in feasibility studies and/or in the validation process.

FFPE is the most common method for preservation of tissue in clinical testing. FFPE tissue is extremely stable and is stored at room temperature for decades with integrity (18). For these reasons, FFPE tissue is the most common specimen type used for routine histopathologic examinations. Studies have shown that the extraction of protein from FFPE tissue for LC–MS analysis is highly successful and does not require overly specialized equipment (19, 20). Comparisons between the proteome of matched FFPE and fresh-frozen tissue specimens have shown minimal variation (21, 22).

**Fig. 3:**
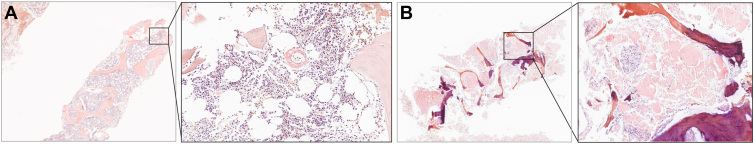
**Bone marrow amyloidosis case images demonstrate the differences in the extent of amyloid deposition.***A,* images of amyloid deposition in a single vessel wall within a bone marrow biopsy. Amyloid protein identification by LC–MS with laser microdissection for enrichment of the amyloid deposits makes it possible to determine the subtype from this focal area of deposition. *B,* in contrast, another bone marrow biopsy in which most of the healthy marrow has been displaced by amyloid deposition.

Amyloidosis diagnosis is made by histopathologic examination of an FFPE tissue biopsy, as previously described, so this specimen type is readily available for amyloid typing. FFPE tissue is compatible with LMD, which allows for enrichment of the amyloid deposits from tissue biopsies. The extent of amyloid deposition in a biopsy varies from focal deposition in the walls of small vessels to extensive replacement of healthy tissue (Fig. 3). A comparison of gross dissection using a needle and LMD of amyloid deposits from tissue resulted in the collection of substantial amounts of nonaffected (healthy) tissue along with the amyloid material. In most cases, the proportion of healthy tissue vastly exceeded that of the amyloid deposits. The presence of a complex mixture of healthy and amyloid-containing tissue resulted in complex proteomic profiles that were difficult to interpret (data not shown). LMD was adopted as a targeted enrichment strategy to isolate regions of interest with high specificity.

A common tissue biopsy in suspected AL amyloidosis cases is bone marrow tissue. Bone and bone marrow specimens routinely undergo decalcification following fixation so that the bone can be cut thinly for histological evaluation. Decalcification is achieved either by acid solutions or a gentler approach, utilizing a chelating agent such as EDTA (23). Comparison testing determined that FFPE tissue samples decalcified using either method were compatible with the downstream LC–MS analysis (data not shown). FFPE tissue offered a readily available and highly compatible specimen type requiring no modifications to existing clinical specimen collection workflows for the amyloidosis typing assay.

#### Protocol Development

Sample preparation optimization was completed prior to validation to avoid repeating validation experiments and will not be explored in depth. This process included maximizing protein extraction from the source material by testing buffers, homogenization methods, protein digestion procedures, and peptide desalting products. A major consideration was reagent compatibility with LC–MS. After evaluations of multiple buffers, a low-concentration zwitterionic detergent in a Tris buffer (pH 8) was chosen. This buffer was stable over extended periods at room temperature, could be frozen if needed, was compatible with endoproteinase digestion, and only required a desalting step prior to injection on the instrument.

Clinical assay preanalytical steps must be highly reproducible across time, staff, and environmental conditions. The amyloid protein identification by LC–MS assay is performed on low tissue amounts, and therefore, to limit sample loss, specimen transfer steps were minimized. Another consideration in preanalytical process development is the clinically acceptable turnaround time (TAT) from test ordering to resulting. Amyloid typing was not a stat test and does not require a rapid turnaround.

One element of protocol development that became very important in recent years is the identification of reliably available reagents and consumables and alternatives in case of shortages or discontinuation. Difficulty obtaining required supplies can significantly impact the stability and sustainability of a clinical assay. Clinical laboratories struggled to procure necessary supplies related to the coronavirus disease pandemic supply chain issues (24, 25). Although that was an unprecedented period, determining the volume of reagents and consumables required for the anticipated volume in the preplanning stages can assist with inventory management. If a reagent or consumable is very specialized and only available from a single vendor, supply chain issues have more drastic impacts on the test offering. Supply chain and inventory management considerations should be part of test development.

LC–MS method development included elution gradient optimization. A comparison of results between a 40-min and 70-min gradient determined that the results were concordant, so the shorter analysis was utilized. EASY Spray 50 cm columns were evaluated and found to be robust, lasting for 1 to 3 months with hundreds of injections. In addition, EASY Spray columns are plug-and-play, which made it easier for clinical laboratory technologists with limited LC–MS experience to quickly master. As discussed, the MS method was a top 10 DDA method with dynamic exclusion of 20 s based on the results of previously performed instrument optimization exercises.

For the amyloid protein identification assay, raw files were searched using Byonic from ProteinMetrics within Proteome Discoverer. Proteome Discoverer provides an interface that was simple to view, rearrange, filter, and navigate for a clinical laboratory technologist with less experience interpreting LC–MS data. Byonic was selected as the algorithm for data analysis because of the high number of variable modifications that can be included in a single search and the ability to define modifications as rare or common (26). Several amino acid modifications can occur during formalin fixation, including formylation and methylation. Most of these modifications occur at less than 5% occupancy on the sites specified, but these were kept in the search parameters to capture a more complete image of the specimen (22, 27, 28). An analysis template was created in Proteome Discoverer to ensure all files were searched using identical parameters and a database.

The first step in test data evaluation is confirming that the total number of protein groups is 50 or more. Amyloid deposit proteomes are not highly complex, and between 50 and 300 protein groups were identified in pilot studies that included different tissues and amyloid types. Protein groups contain groups that cannot be unambiguously identified by unique peptides in the search output. The percentage of PSMs with a missed cleavage must be 30% or less for all identified protein groups. If the percentage of the missed cleavage is higher, it could indicate an issue with the trypsin enzyme, including interference or the enzyme quality.

The protein identification by LC–MS assay is qualitative but utilizes spectral counting to determine relative quantitation of proteins in the amyloid deposits. Liu *et al.* (29) demonstrated that the number of MS/MS spectra assigned to a protein, spectral counts, correlate linearly with the protein's relative abundance across more than two orders of magnitude in a complex mixture. Although this is an effective technique, it must be said that larger proteins generally generate more peptides, and there is an increased likelihood of MS/MS spectra of peptides from larger proteins. However, generally, spectral counting can provide reliable relative abundance information within a sample. For amyloid subtype determination, this is an effective technique because the amyloid deposits are isolated from surrounding tissue, simplifying the proteome.

As outlined in the Experimental procedures section, APOE and SAMP were identified with five or more PSMs in the positive amyloid specimen. APOE and SAMP are found localized to amyloid deposits along with the causative or subtype protein(s). APOE is most discussed because of its variants' relationship to Alzheimer's disease and other amyloid neurodegenerative diseases. This protein is most highly expressed in endocrine tissue, the brain, and skin (30). SAMP has been shown to bind to all types of amyloid fibrils and is theorized to protect them from degradation (31). This protein also serves as a versatile immunomodulatory factor that is expressed by hepatocytes and can be found in blood.

Amyloid disease type could be determined if the subtype protein(s) have five or more PSMs, and all other known subtype proteins are not detected or present with less than five PSMs. A case is determined to be inconclusive for subtype if results meet APOE and SAMP requirements, but a subtype cannot be conclusively determined based on the previously stated requirements. A specimen is negative for amyloidosis if APOE and SAMP are not detected or with less than five PSMs.

### Feasibility Studies

#### Interference and Limitations

Interference comes from other analytes naturally occurring in the specimen or are introduced in the sample processing steps. For the amyloid protein identification assay, if all established preanalytical protocols are followed, no interfering substances from the sample matrix have been observed. However, as previously discussed, if LMD is not performed, healthy tissue regions are captured the nonamyloid components will interfere with the interpretation of the results.

#### Assay Controls

Healthy myometrium tissue was selected as a negative control, and a previously characterized positive ATTR lung biopsy was selected as a positive control. The control samples were prepared in parallel with the patient specimen. These serve as process controls and sensitivity and specificity controls. Positive controls for the assay could be any FFPE tissue with amyloid deposition with a known subtype and negative controls could be any FFPE tissue specimen without amyloid deposition. Healthy myometrium was selected as it was readily available at the time of development. As amyloidosis may occur in nearly any tissue or organ, controls are not limited to specific FFPE tissue types.

### Analytical and Clinical Validation

#### Reportable Range

The reportable range for an assay is the span of values over which a test or instrument can produce a result that is directly proportional to the amount of the analyte. Reportable range is not applicable to the amyloid protein identification test, as the assay is qualitative and results are reported as positive for a specific subtype, inconclusive for subtype, or negative for amyloidosis. A strict set of criteria was established for the positive samples, including a minimum of 50,000 μm^2^ of Congo red–positive tissue for each replicate, with three replicates as the goal. At least two replicates were processed for all validation samples. Negative controls had the same minimum tissue and replicate requirements. For all samples, one-fifth of the final peptide digest was analyzed by LC–MS. The other general criteria were the minimum number of five PSMs identified from the two general amyloidosis biomarkers, APOE and SAMP, in at least two of the three replicates, which all specimens achieved.

#### Accuracy

The accuracy of a clinical test is optimally determined by comparing the results to an existing approved assay. This can be accomplished by sending specimens out to a reference laboratory that offers a comparable assay, but this is not always possible because of a lack of assay availability. Comparisons of results with a different method for the detection of the biomarker(s) are acceptable. For example, IHC or other antibody-based methods can be used for comparison to results from a protein MS-based method. As outlined in Table 1, the accuracy of the amyloid protein identification by LC–MS assay was 100% when applied to a highly diverse set of FFPE tissue.

#### Precision

Precision is a measure of repeatability of a measurement or if the assay and/or instrument gives the same result when testing the same sample repeatedly. Precision does not indicate that the result is correct but measures the random error of the method. Intrarun or within-run precision will indicate the expected daily performance of the assay and the instrument. To determine intrarun precision of the test, the same sample(s) is measured multiple times in a single run, batch, or day, depending upon the way the test is performed. Between-run or inter-run precision involves testing the same sample multiple times across different days and comparing the results across those separate days. Inter-run or interday precision measures the variation because of changes in operating conditions, including laboratory environmental conditions and personnel. Both interday and intraday precision were assessed by injecting the same amyloid specimen twice per day on 4 days during a 7-day period. The number of PSMs for APOE, SAMP, and subtype protein(s) was used to determine that the relative variance was less than 20% for all three of the specimens involved.

#### Analytical Sensitivity

Analytical sensitivity is the lowest or smallest amount of an analyte that an assay can accurately measure or the limit of detection. Prior to validation, optimization experiments determined that a minimum quantity of 50,000 μm^2^ of Congo red–positive tissue is required to reproducibly perform testing. Although a fifth of the samples is analyzed by LC–MS, this minimum tissue is required because of sample losses during preparation. If less tissue is collected during LMD, the results are not reproducible. Therefore, the lower end of the analytical sensitivity of the assay is set at 50,000 μm^2^ of tissue. All LMD-prepared samples in the validation cohort contained a minimum of 50,000 μm^2^, meaning that analytical sensitivity was 100% based on the established guidelines of the assay.

#### Analytical Specificity

Analytical specificity is the assay's ability to detect the analyte(s). This requires assessment of crossreactivity and interference from endogenous and exogenous sources. Endogenous substances may include other proteins naturally in the specimen or medications the patient is taking. Exogenous sources of crossreactivity or interference could come from sample additives, collection tubes, or other consumables used. In addition, abnormal sample conditions because of differences in sample handling or coexisting patient conditions should be assessed to fully evaluate interference. Analytical specificity was the amyloid protein identification by LC–MS assay was 100% as APOE, SAMP, and amyloid subtype protein(s) were only detected in the positive specimen.

#### Clinical Sensitivity

Clinical sensitivity is the ability of the test to identify the presence of a disease, illness, or abnormality correctly and is associated with the numbers of true positives and false negatives. True positives are when a patient has an illness or disease, and the test gives a positive result. A false negative is when a patient has an illness or disease, and the test result is negative. The proportion of true positives to all positives, which would be true positives plus false negatives, provides a value for clinical sensitivity. For example, if there are 20 patients with the disease, 18 patients get a positive result, and 2 patients get a negative result, the clinical sensitivity is 90%. As discussed, the clinical sensitivity was 100% for this assay. All cases that were confirmed to be positive for amyloidosis by Congo red staining with a pathologist met the requirements to be called positive by LC–MS.

#### Clinical Specificity

Clinical specificity is the ability of the assay to identify the absence of a disease or illness correctly. Otherwise stated, it is the ability to obtain normal range or negative results for a specimen from a patient without the disease. Specificity values are related to the number of true negatives and false positives. True negatives are when the specimen from a patient without the disease gives a negative or normal result, and a false positive is when the test result is positive or abnormal for a patient without the disease. Specificity can be determined by evaluating the ratio of true negatives to the total real-negative specimen. If there are again 20 patients without the disease, 19 patients are true negatives, and 1 is a false negative, then the specificity is 95%. A higher specificity is more likely to give a true negative result. All Congo red–negative cases were confirmed negative by LC–MS typing.

### Documentation and Standard Operating Procedure Finalization

#### Expected Results, Acceptance, Rejection Criteria, and Troubleshooting

Once the validation was complete, all data were compiled and reviewed for submission to the New York State Clinical Laboratory Evaluation Program. Clear criteria for test interpretation were set for the amyloid protein identification assay, starting with the evaluation of instrument QC results, followed by batch control results (Supplemental Table S6). Amyloid positive control and negative control results must meet the previously outlined requirements for protein groups, number of PSMs for amyloid and subtype biomarkers, and % missed cleavage. Finally, the patient specimen results are interpreted. For each patient case, a blank injection of only mobile phase A is run and analyzed using the same LC–MS method prior to the patient specimen to determine if there was any sample carryover. The blank data follow the same data analysis and interpretation workflow as the patient specimen. If there is evidence of carryover from the previous patient specimen in the blank, the patient samples are reanalyzed on the instrument.

For patient samples, at least two of three replicate results must be concordant. If there are fewer than three replicates, all must be concordant. A case is inconclusive for subtype if results meet APOE and SAMP requirements, but a subtype cannot be conclusively determined. A specimen is reported as negative for amyloidosis if fewer than five PSMs for APOE and SAMP are reported. This can occur if there is nonspecific Congo red staining that is misinterpreted as positive, although none of these types of cases were included in the validation cohort.

A comprehensive set of troubleshooting guidelines were developed to address potential issues that may arise during assay execution. QC and troubleshooting procedures cover all critical stages of the assay, including sample preparation, instrument performance, data acquisition, and analysis. For each step, specific corrective actions are outlined to resolve failures, minimize disruptions, and ensure the continued reliability and integrity of the testing process. This structured approach enables timely identification and resolution of problems, supports compliance with clinical laboratory standards, and ensures consistent, high-quality test results.

#### Standard Operating Procedure Catalog Finalization

A Standard Operating Procedure catalog was written to not only cover the amyloid protein identification by LC–MS assay sample preparation, analysis, data interpretation, and reporting but also includes procedures for maintenance, QC, operation, and troubleshooting of instrumentation and equipment. All SOPs were written so that someone with basic clinical laboratory experience could follow.

#### QC Plan

In addition to assay controls, a well-defined instrument QC plan for assessing instrument performance was developed. This included system maintenance procedures and schedules. Following any maintenance, including mobile phase or column replacements, QC standards, including the peptide retention mix and HeLa lysate digest, were analyzed and evaluated as outlined previously and tracked on in-house built electronic forms. These forms are used to record QC standard results and enable workflows for supervisory review (Supplemental Fig. S1).

Daily monitoring of the LC–MS performance, including sensitivity, mass accuracy, and fragmentation efficiency, is critical. A retention time peptide standard is a good option and has worked well in clinical workflows. Some examples are the peptide retention time calibration mixture from Thermo Scientific, Promega 6 × 5 LC–MS/MS Peptide Reference Mix, or the iRT from Biognosys, but there are others available commercially. These types of standards contain a selection of synthetic peptides that will not be naturally found in proteomic samples because they are heavy isotope labeled or non-naturally occurring peptides. This type of standard is used to evaluate both the LC and MS components. This simple yet complete system evaluation is performed at least once every 24 h to ensure instrument performance is optimal. Instrument downtime and patient result delays are minimized by early detection of instrument performance problems. A monthly Levey–Jennings chart of the PRTC peptide intensities provides a longitudinal review of instrument performance.

### Approval and Implementation

#### Test Ordering, Billing, and Reporting

The completed validation packet was submitted to the New York State Clinical Laboratory Evaluation Program, and once approved, preparation was made to start patient testing. A critical part of implementation was determining the processes for test ordering, reporting, and billing. Many tests are ordered directly by clinical care team members in electronic health systems. Other assays are considered reflex tests and are automatically added on depending upon the results of other tests. Alternatively, some tests are manually ordered if a technologist, pathologist, or other specialists determines they are required based following a review of other test results.

The amyloid protein identification by LC–MS assay at our institution is only ordered if requested by a pathologist upon review of the specimen. If there is clinical suspicion for amyloidosis or a pathologist identifies amorphous deposits in a biopsy, a Congo red stain is ordered. If that stain is positive, then the pathologist can request the amyloid typing assay. The pathologist can consult with the laboratory director to determine if the specimen is compatible with testing. There is no process for clinicians to directly order the testing, as it is based on other histological findings. Education of the clinical care teams, pathologists, and other individuals responsible for test requests regarding the utility of the test and clinical significance of results is also required. Not insignificantly, appropriate billing codes must be assigned and associated within the test ordering system.

Once a test is ordered and completed, the results must be reported in the patient's electronic health record. For amyloid protein identification by LC–MS, the data are initially evaluated and interpreted by a technologist using the protein group, missed cleavage, and PSM requirements outlined. Next, the data summary is evaluated by the laboratory director within the clinical context of the patient and then reviewed the results with a molecular pathologist who has had appropriate training. Results are then verified by the pathologist and appear in a patient's medical record for the clinical team and patient to review. For example, if a specimen is positive for amyloid, the report may read “the amyloid protein identification by LC–MS/MS test identified a peptide profile consistent with AL lambda amyloid deposition.” Standardized text was established for when a specimen is positive, negative, or inconclusive to ensure the results are clearly stated and consistent over time (Supplemental Fig. S2).

Results for the amyloid protein identification by LC–MS test are expected within 14 analytical days, which means within 14 operational or business days. If results are not reported within that time frame, then the laboratory did not meet the defined TAT. TAT is an important metric in the QA plan because patients and clinical care teams will expect results within the set TAT window. Any delays in reporting are monitored and recorded. Systemic issues can be addressed to minimize TAT delays. Tracking TAT is a key performance indicator used to evaluate the overall laboratory performance.

#### Training

Staff readiness and training are a critical part of test implementation. Training plans were developed, including training checklists that document completion of training prior to testing patient specimen. Staff training plans for the amyloid protein identification by LC–MS include lectures, observation of the assay performance, execution of the testing procedures under supervision, and finally, end-to-end performance of the test procedure on a set of known assessment specimens. Additional training occurs if SOPs change or errors are observed.

### Compliance, Monitoring, and Refinement

Validation and implementation of an LDT is just the beginning of the process, as the laboratory must continue to meet requirements, including inspections, quality system monitoring, analytical system monitoring, and accuracy and reliability of testing. Once the amyloid protein identification by LC–MS test was live, continuous monitoring of quality, performance, and compliance was put into place.

#### Staff Competency Assessments

All staff have annual competency assessments where laboratory supervisory staff observe team members performing their clinical laboratory duties. During these observations, the supervisors determine if the staff member is following all procedures, guidelines, and regulations. If competency is not met in any area, an action plan is designed to provide developmental activities for that team member to meet competencies.

#### Proficiency Testing

Biannual proficiency testing is required to evaluate the laboratory's ability to perform the assay. Generally, proficiency testing is when a laboratory analyzes unknown samples to ensure accurate testing results. For common clinical tests, an external party provides proficiency testing samples. However, for some LDTs, an internal proficiency procedure will be followed as no external proficiency program exists. Internal proficiency can involve retesting of previously tested material or in-house developed standards. For the amyloid protein identification by LC–MS test, no external proficiency program currently exists. The current internal proficiency testing procedure is that every 6 months, three specimens that have been tested within the past 6 to 12 months are retested. Results are assessed to ensure they meet all established requirements and are concordant with the initial testing results.

#### Inspections

As discussed, in the United States, CLIA-licensed laboratories undergo regular inspections by approved accrediting organizations to ensure CLIA compliance. During inspections, adherence to regulations is evaluated, and deficiencies may be discovered. Even if there are no findings requiring action, the inspectors may make recommendations for improvements to quality systems or operational procedures. Continuous evaluation of laboratory procedures is essential to maintaining a reliable testing environment. This is why quality and process improvement projects and regular staff training and continuing education are part of standard operations in clinical laboratories. In addition, internal audits performed by institutional regulatory affairs and quality assurance teams are important to maintain reliable testing.

#### Assay Modifications

Disulfide bond reduction and alkylation are common in proteomics workflows and have been shown to increase the efficiency of the enzyme digestion and increase sequence coverage (32). Structural studies of light chain amyloid fibrils by cryo–electron microscopy indicated that intramolecular disulfide bonding occurs (33). For these reasons, reduction and alkylation were assessed, validated, and added to the specimen processing for the assay. The additional steps did not largely impact the test TAT and resulted in a 40% increase in light chain sequence coverage.

Feasibility and validation studies were performed using DIRECTOR slides for LMD, but the company that produced the slides was sold, and it became difficult to order the slides. For that reason, different slide types were evaluated to identify a backup. It was clear that with the LMD system, only the PEN membrane slides performed comparably to the DIRECTOR slides. In general, PEN membrane slides are recommended for use with UV laser–equipped LMD, and results of the testing proved the slide type to be compatible with the assay (20, 34). In the case of infrared laser LCM systems, a PEN membrane or uncharged slide should work (34). Validating the PEN membrane slides was critical to maintaining test continuity once the DIRECTOR slides became unavailable and our stock was depleted.

### Other Approaches to Amyloid Typing With MS

The amyloid protein identification by LC–MS test in our laboratory is a qualitative (semiquantitative) assay. DDA and relative spectral counts are used to determine the most abundant proteins in the specimen. LMD isolates amyloid deposits, which have a simpler proteome than the surrounding healthy tissue. Therefore, causative or subtype protein(s) should be among the most abundant. Many institutions in the United States and internationally have validated and implemented a version of this assay. The majority have been similar with the use of LMD and DDA acquisition.

However, as MS technology evolves, other clinical laboratories have explored different approaches to amyloid characterization. Some have explored the use of targeted MS but were limited to the number of subtypes that could be confidently detected (35, 36). As discussed, there are more than 30 subtypes of amyloidosis, caused by different proteins. Developing a targeted quantitative method for all potential subtypes, including known hereditary mutations, would still exclude unknown subtypes and variants. A targeted assay could identify transthyretin amyloidosis but would not be able to differentiate wildtype from hereditary. There are currently over 100 different reported amyloidogenic mutations that have been identified.

At the time of our assay, development and validation data-independent acquisition (DIA) techniques and data analysis tools were not as advanced. DIA MS data analysis depended upon well-characterized spectral libraries, which would have posed similar problems as targeted analysis for novel subtypes or variants. Even many known subtypes are not frequently observed at institutions, making it difficult to create an inclusive spectral library. However, DIA data analysis techniques and tools have expanded, allowing for searches against sequence databases or *de novo* sequence determination (37). DIA acquisition has been shown to provide similar results than DDA in shorter analysis times (38).

We determined that LMD is essential to accurately and reproducibly determine the amyloid subtype. However, LMD equipment is expensive and requires training on tissue morphology. For these reasons, efforts have been made to develop assays that do not rely upon microdissection of tissue and instead rely upon the expertise of pathologists to manually dissect the deposits from tissue sections using a dissection microscope (39). This approach was successful at discriminating between the major amyloid subtypes by employing a normalized spectral abundance factor system to compare protein abundances. A MALDI MS imaging method was also developed for amyloid protein identification, which would not require any removal of tissue (38, 40). These diverse efforts highlight the benefit that LDTs can be developed utilizing the instrumentation and resources available at different institutions.

## Conclusion

**Fig. 4:**
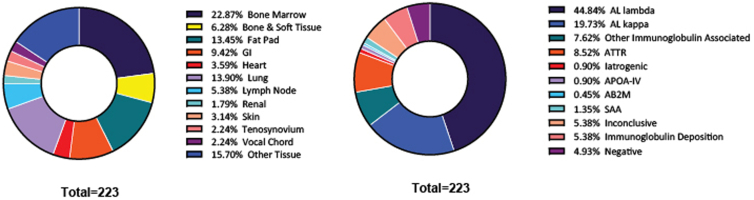
**Amyloid cases analyzed since assay implementation.** Since the implementation of the amyloid protein identification by LC–MS assay, a total of 223 cases have been reported in patient health records. These cases have included over 11 different tissue types tested and over nine different subtypes, including a novel iatrogenic form.

From the implementation of the amyloid protein identification by LC–MS assay in our laboratory till the end of 2024, 223 clinical cases have been reported (Fig. 4). Utilization of this LDT at our institution has allowed for the discovery of a novel iatrogenic form of amyloidosis caused by liraglutide injections (41). This assay was also used to characterize the proteomic profile of localized cutaneous amyloidosis, for which the causative protein is still under debate (42). In addition, a study into rare cases of mammary amyloidosis was conducted with this technology (43). Most importantly, the assay provides patients suffering from this rare group of related diseases with access to a diagnostic test that enables accurate classification and supports appropriate, targeted treatment.

This work is conducted in close collaboration with clinicians, pathologists, and other subject matter experts to ensure the assay is developed within the appropriate clinical context and in full compliance with regulatory standards. Through this collaborative and disciplined approach, we aim to advance reliability, clinical utility, and broader adoption of specialized diagnostic tools, such as the amyloid protein identification assay.

LDTs require expertise, careful planning, rigorous evaluation, and thoughtful implementation. Amyloid typing by LC–MS has shown that a tissue LC–MS proteomics test can have a significant impact on the diagnosis and care of patients. Identification of the causative protein allows for confident diagnosis and proper treatment. Implementation of variations of this LDT has been critical for diagnostic and treatment workflows internationally. There are consortia of medical personnel, scientists, and others who have formed networks that provide guidance for others looking to provide this essential gold-standard testing. Amyloid typing has been the first major LC–MS–based proteomics assay implemented in a molecular pathology setting and highlights the potential for further utility.

## Data Availability

Data are available *via* Proteome Exchange PXD062691.

## Supplemental Data

This article contains supplemental data.

## Conflict of Interest

The authors declare no competing interests.

## Figures and Tables

**Figure s1:**
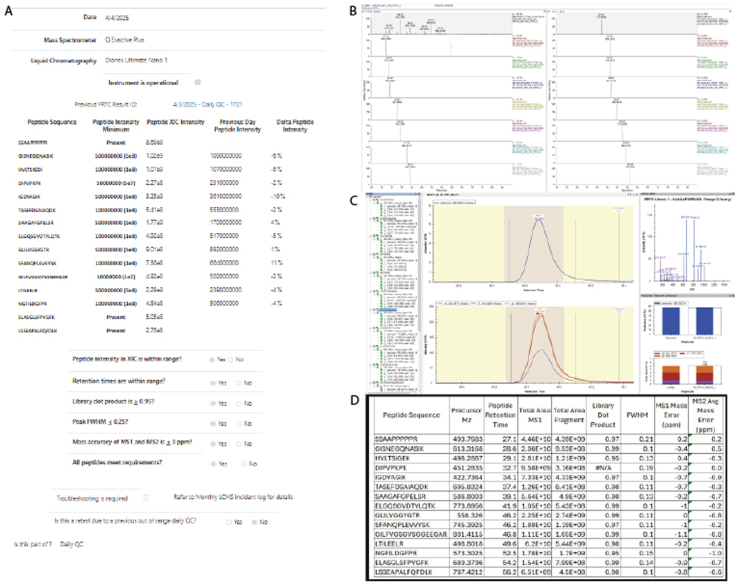
**Electronic daily QC log contains the PRTC peptide mixture key performance indicators and allows for instrument performance tracking.***A,* daily log with peptide intensities and % change in intensity is shown. *B,* the base peak chromatogram and extracted ion chromatograms are reviewed for all PRTC peptide precursors. *C,* Skyline is used to evaluate mass accuracy, peak width, peak intensity, and to compare fragmentation patterns. *D,* data from Skyline can be easily exported to an Excel document, evaluated, and attached to the electronic log for later reference. PRTC, peptide retention time calibration mixture; QC, quality control.
