# Supplementary material for: Validation of a Mass Spectrometry–Based Proteomics Molecular Pathology Assay
Source: Mol Cell Proteomics. 2025 Dec 12;25(1):101487. doi: 10.1016/j.mcpro.2025.101487 (PMC12854024; doi:10.1016/j.mcpro.2025.101487)
Supplement: Figure S2 [file mmc1.docx]

**Figure S2.** Result report templates were created for the Amyloid Protein Identification by LC-MS assay to ensure language is clear and consistent. Additional comments or important information is added as required.

**Example 1.**

**Addendum Diagnosis**

The amyloid protein identification by LC-MS/MS test identified a peptide profile consistent with AL lambda amyloid deposition.

**Addendum Comment**

Amyloid Protein Identification by LC-MS/MS:

Test Methodology: Amyloid protein identification by laser microdissection of Congo red stained tissue on a DIRECTOR or PEN membrane slide and liquid chromatography-tandem mass spectrometry (LC-MS/MS).

Test Interpretation: LC-MS/MS based proteomic analysis identified a peptide profile consistent with AL lambda type amyloid deposition.

**Example 2.**

**Addendum Diagnosis**

The amyloid protein identification by LC-MS/MS test was NEGATIVE for a peptide profile associated with amyloidosis. Testing of another biopsy or site is recommended to establish amyloid diagnosis and classification.

**Addendum Comment**

Amyloid Protein Identification by LC-MS/MS:

Test Methodology: Amyloid protein identification by laser microdissection of Congo red stained tissue on a DIRECTOR or PEN membrane slide and liquid chromatography-tandem mass spectrometry (LC-MS/MS).

Test Interpretation: LC-MS/MS based proteomic analysis was NEGATIVE for a peptide profile associated with amyloidosis.

**Example 3.**

**Addendum Diagnosis**

The amyloid protein identification by LC-MS/MS test was positive for a peptide profile consistent with amyloidosis but was inconclusive with regards to amyloid type. Testing of another biopsy or site is recommended to establish amyloid diagnosis and classification.

**Addendum Comment**

Amyloid Protein Identification by LC-MS/MS:

Test Methodology: Amyloid protein identification by laser microdissection of Congo red stained tissue on a DIRECTOR or PEN membrane slide and liquid chromatography-tandem mass spectrometry (LC-MS/MS).

Test Interpretation: LC-MS/MS based proteomic analysis identified a peptide profile consistent amyloidosis but was inconclusive with regards to amyloid-type.
