# Supplementary material for: Validation of a Mass Spectrometry–Based Proteomics Molecular Pathology Assay
Source: Mol Cell Proteomics. 2025 Dec 12;25(1):101487. doi: 10.1016/j.mcpro.2025.101487 (PMC12854024; doi:10.1016/j.mcpro.2025.101487)
Supplement: Figure S3 [file mmc2.docx]

| Accession # | Protein Name | Gene Name | # Peptide Spectral Matches | | |
| --- | --- | --- | --- | --- | --- |
|  |  |  | Replicate 1 | Replicate 2 | Replicate 3 |
| P02766 | Transthyretin* | TTR | 34 | 22 | 25 |
| P02743 | Serum Amyloid P** | APCS | 20 | 23 | 25 |
| P01024 | Complement C3 | C3 | 24 | 22 | 26 |
| P68871 | Hemoglobin beta | HBB | 45 | 24 | 11 |
| P69905 | Hemoglobin alpha | HBA | 32 | 21 | 12 |
| P04004 | Vitronectin | VTN | 19 | 20 | 16 |
| P06727 | Apolipoprotein A-IV | APOA4 | 19 | 16 | 23 |
| P02649 | Apolipoprotein E** | APOE | 10 | 10 | 8 |
| P02768 | Serum albumin | ALB | 29 | 21 | 20 |

| Accession # | Protein Name | Gene Name | # Peptide Spectral Matches | | |
| --- | --- | --- | --- | --- | --- |
|  |  |  | Replicate 1 | Replicate 2 | Replicate 3 |
| P0CG05 | Ig lambda chain C2* | IGLC2 | 331 | 310 | 317 |
| P12111 | Collagen alpha-3 chain | COL6A3 | 33 | 98 | 51 |
| P04004 | Vitronectin | VTN | 29 | 25 | 24 |
| P02649 | Apolipoprotein E** | APOE | 26 | 37 | 29 |
| P02748 | Complement C9 | C9 | 25 | 16 | 21 |
| P01703 | Ig Lambda V-1 region NEWM* |  | 23 | 27 | 25 |
| P10909 | Clusterin | CLU | 18 | 24 | 19 |
| P02647 | Apolipoprotein A-I | APOA1 | 19 | 18 | 21 |
| P02743 | Serum Amyloid P** | APCS | 10 | 12 | 15 |

**Figure S3.** Search results are interpreted to determine if the specimen meets the requirements to be called positive for amyloidosis which requires the detection of serum amyloid P protein and apolipoprotein E. If the results confirm the specimen is positive for amyloidosis the subtype is determined by considering the most abundant proteins present in the sample. (A) AL amyloidosis is caused by an immunoglobulin light chain and high levels of immunoglobulin constant region and variable region for either lambda or kappa will be detected. (B) ATTR amyloidosis is caused by either wild type or mutated transthyretin protein, and it is one of the most abundant proteins detected in the amyloid deposits from patients with ATTR.
