# Supplementary material for: Validation of a Mass Spectrometry–Based Proteomics Molecular Pathology Assay
Source: Mol Cell Proteomics. 2025 Dec 12;25(1):101487. doi: 10.1016/j.mcpro.2025.101487 (PMC12854024; doi:10.1016/j.mcpro.2025.101487)
Supplement: Table S2 [file mmc4.docx]

Table S2. Reduction and alkylation of amyloid tissue samples is required to increase sequence coverage of subtype proteins specifically immunoglobulins.

| **Patient #** | **Rep#** | **Red/Alk** | **# Protein Groups**  > 100 | **# PSMs APOE**  + = > 5  - = < 5 | **# PSMs SAMP**  + = > 5  - = < 5 | **# PSMs**  **Ig κ**  + = > 5  - = < 5 | **# PSMs**  **Ig λ**  + = > 5  - = < 5 | **Amyloid Subtype** | **Meets Requirements** | **Concordant?** |
| --- | --- | --- | --- | --- | --- | --- | --- | --- | --- | --- |
| 1 | A | NO | 178 | 36 | 28 | 22 | 0 | AL kappa | YES | YES |
| 1 | B | NO | 210 | 38 | 25 | 33 | 2 |  | YES | YES |
| 1 | C | YES | 176 | 31 | 13 | 47 | 1 |  | YES | YES |
| 1 | D | YES | 172 | 43 | 21 | 46 | 1 |  | YES | YES |
| 2 | A | NO | 225 | 11 | 11 | 44 | 0 | AL kappa | YES | YES |
| 2 | B | NO | 185 | 21 | 5 | 49 | 0 |  | YES | YES |
| 2 | C | YES | 163 | 19 | 7 | 75 | 1 |  | YES | YES |
| 2 | D | YES | 115 | 10 | 7 | 48 | 0 |  | YES | YES |
| 3 | A | NO | 179 | 20 | 13 | 9 | 33 | AL lambda | YES | YES |
| 3 | B | NO | 185 | 41 | 10 | 9 | 34 |  | YES | YES |
| 3 | C | YES | 110 | 23 | 14 | 6 | 25 |  | YES | YES |
| 3 | D | YES | 121 | 28 | 20 | 6 | 26 |  | YES | YES |
