# Supplementary material for: Validation of a Mass Spectrometry–Based Proteomics Molecular Pathology Assay
Source: Mol Cell Proteomics. 2025 Dec 12;25(1):101487. doi: 10.1016/j.mcpro.2025.101487 (PMC12854024; doi:10.1016/j.mcpro.2025.101487)
Supplement: Table S3 [file mmc5.docx]

Table S3. Reduction and alkylation of amyloid tissue samples is required to increase sequence coverage of subtype proteins specifically immunoglobulins.

| **Patient #** | **Rep#** | **Red/Alk** | **Amyloid Subtype** | **% Coverage APOE** | **% Coverage SAMP** | **% Coverage Ig κ** | **% Coverage Ig λ** |
| --- | --- | --- | --- | --- | --- | --- | --- |
| 1 | A | NO | AL kappa | 61 | 43 | 49 | 0 |
| 1 | B | NO |  | 63 | 41 | 49 | 5 |
| 1 | C | YES |  | 60 | 35 | 85 | 14 |
| 1 | D | YES |  | 57 | 36 | 80 | 14 |
| 2 | A | NO | AL kappa | 32 | 38 | 56 | 0 |
| 2 | B | NO |  | 61 | 19 | 56 | 0 |
| 2 | C | YES |  | 51 | 23 | 100 | 14 |
| 2 | D | YES |  | 34 | 28 | 91 | 0 |
| 3 | A | NO | AL lambda | 39 | 35 | 49 | 32 |
| 3 | B | NO |  | 59 | 23 | 49 | 53 |
| 3 | C | YES |  | 62 | 29 | 52 | 70 |
| 3 | D | YES |  | 58 | 34 | 65 | 86 |
