# Supplementary material for: Validation of a Mass Spectrometry–Based Proteomics Molecular Pathology Assay
Source: Mol Cell Proteomics. 2025 Dec 12;25(1):101487. doi: 10.1016/j.mcpro.2025.101487 (PMC12854024; doi:10.1016/j.mcpro.2025.101487)
Supplement: Table S4 [file mmc6.docx]

Table S4. Tissue recovery is higher when performing laser microdissection with a UV laser system off of DIRECTOR or PEN membrane slides as compared to glass.

| **Slide Type** | **# Protein Groups**  > 100 | **# PSMs APOE**  + = > 5  - = < 5 | **# PSMs SAMP**  + = > 5  - = < 5 | **# PSMs TTR**  + = > 5  - = < 5 | **# PSMs SAA**  + = > 5  - = < 5 | **# PSMs**  **Ig κ**  + = > 5  - = < 5 | **# PSMs**  **Ig λ**  + = > 5  - = < 5 | **Meets Requirements** | **Concordant?** |
| --- | --- | --- | --- | --- | --- | --- | --- | --- | --- |
| Uncharged | 66 | 2 | 1 | 0 | 0 | 0 | 8 | NO | NO |
| Charged | 65 | 4 | 2 | 1 | 0 | 0 | 17 | NO | NO |
| DIRECTOR | 181 | 22 | 10 | 3 | 0 | 3 | 28 | YES | YES |
| PEN membrane | 184 | 21 | 7 | 4 | 0 | 4 | 37 | YES | YES |
