# Supplementary material for: Validation of a Mass Spectrometry–Based Proteomics Molecular Pathology Assay
Source: Mol Cell Proteomics. 2025 Dec 12;25(1):101487. doi: 10.1016/j.mcpro.2025.101487 (PMC12854024; doi:10.1016/j.mcpro.2025.101487)
Supplement: Table S5 [file mmc7.docx]

Table S5. Amyloid protein identification by LCMS test results are concordant when tissue is laser microdissected from PEN membrane slides or DIRECTOR slides.

| **Sample** | **Slide Type** | **# Protein Groups**  > 50 | **# PSMs APOE**  + = > 5  - = < 5 | **# PSMs SAMP**  + = > 5  - = < 5 | **# PSMs TTR**  + = > 5  - = < 5 | **# PSMs SAA**  + = > 5  - = < 5 | **# PSMs**  **Ig κ**  + = > 5  - = < 5 | **# PSMs**  **Ig λ**  + = > 5  - = < 5 | **Meets Requirements** | **Concordant?** |
| --- | --- | --- | --- | --- | --- | --- | --- | --- | --- | --- |
| **P1 – A** | DIRECTOR | 113 | 28 | 19 | 0 | 0 | 7 | 25 | YES | YES |
| **P1 – B** | DIRECTOR | 131 | 26 | 19 | 0 | 0 | 7 | 25 | YES | YES |
| **P1 – C** | DIRECTOR | 134 | 27 | 18 | 0 | 0 | 12 | 25 | YES | YES |
| **P1 – D** | Membrane | 135 | 27 | 27 | 0 | 0 | 15 | 25 | YES | YES |
|  |  |  |  |  |  |  |  |  |  |  |
| **P2 – A** | DIRECTOR | 123 | 10 | 6 | 0 | 0 | 0 | 36 | YES | YES |
| **P2 – B** | DIRECTOR | 116 | 12 | 4 | 0 | 0 | 0 | 43 | YES | YES |
| **P2 – C** | DIRECTOR | 150 | 11 | 5 | 0 | 0 | 0 | 35 | YES | YES |
| **P2 – D** | Membrane | 106 | 8 | 4 | 0 | 0 | 0 | 40 | YES | YES |
|  |  |  |  |  |  |  |  |  |  |  |
| **P3 – A** | DIRECTOR | 183 | 21 | 22 | 23 | 0 | 0 | 0 | YES | YES |
| **P3 – B** | Membrane | 118 | 14 | 16 | 11 | 0 | 0 | 0 | YES | YES |
