# Supplementary material for: Validation of a Mass Spectrometry–Based Proteomics Molecular Pathology Assay
Source: Mol Cell Proteomics. 2025 Dec 12;25(1):101487. doi: 10.1016/j.mcpro.2025.101487 (PMC12854024; doi:10.1016/j.mcpro.2025.101487)
Supplement: Table S6 [file mmc8.docx]

Table S6. PRTC and HeLa QC samples parameter requirements must be met to ensure instrument performance is sufficient and to proceed with patient testing.

| **PRTC QC Parameter** | **Requirement** |
| --- | --- |
| Retention Time (min) | +/- 1.5 min |
| Peak FWHM (min) | < 0.25 |
| Peak Intensity (AU) | Above minimum intensity & < 50% drop in intensity |
| Precursor Mass Accuracy | +/- 3 ppm from theoretical |
| Fragment Ion Mass Accuracy | +/- 3 ppm from theoretical |
| Spectral Library dot product | > 0.95 |

| **HeLa QC Parameter** | **Requirement** |
| --- | --- |
| # Protein Groups | > 1800 |
| % Conversion (MS2 🡪 PSM) | > 45% |
| Base Peak Intensity | > 5e^8^ |
